# Capillary-Assisted Monitoring of Milk Freshness via a Porous Cellulose-Based Label with High pH Sensitivity

**DOI:** 10.3390/foods12091857

**Published:** 2023-04-29

**Authors:** Ruoting Liu, Wenrui Chi, Qihao Zhu, Hailan Jin, Jian Li, Lijuan Wang

**Affiliations:** Key Laboratory of Bio-Based Materials Science and Technology of Ministry of Education, Northeast Forestry University, No. 26 Hexing Road, Xiangfang District, Harbin 150040, China

**Keywords:** bis-quaternary ammonium salt, porous cellulose-based label, capillarity, pH sensitivity, milk freshness

## Abstract

A cellulose-based matrix for monitoring milk freshness (MF) was produced from rice straw particles (RSPs) in a 0.125–0.150 mm that was bis-quaternized to attach bromocresol purple (BP) as a sensor. Under alkali conditions, the obstinate structure of the rice straw had opened, thereby improving the accessibility of the cellulose. Bis-quaternization created more adsorption sites for BP. The maximum adsorption capacity was 97.68 mg/g. The sensors were interwoven with cellulosic fibers to form the cellulose-based label with a relatively loose three-dimensional structure via hydrogen bonds. As the proportion of BP-BCRPs was increased from 10% to 40%, the air permeability of the label increased from 3.76 to 15.01 mm/s, which increased the response to the tested gases (10.12 s for 1 mL of acetic acid). The intelligent label exhibited excellent sensitivity at pH values of 3–9 with highly saturated color changes. During the storage period, the label color shifted from blue-purple to yellow as acidity was increased from 17.24 to 19.8 °T due to capillarity action, providing a timely warning to consumers. The prepared colorimetric porous intelligent cellulose-based label is suitable for monitoring of MF.

## 1. Introduction

Milk is composed of ~87% water, the fat-soluble vitamins A, D, E, and K, proteins (primarily casein and lactalbumin), carbohydrates (primarily lactose), and minerals (including calcium and phosphorus) [1,2,3]. However, milk is highly perishable because of microbial activity and oxidation of proteins, and drinking low-quality milk may cause immeasurable safety risks [4]. Traditional tests of MF consisting of physical-chemical methods (“clot on boiling”, acidity, methylene blue tests, and fatty acid) and microbial growth assays are highly precise, but they require analytical equipment, trained technicians, and time. Particularly, the microbial growth assays need a long incubation period (12 h) [5,6]. Hence, a rapid, sensitive, and simple method is urgently needed to assess MF. In recent years, electrical conductivity is used to provide milk freshness information due to its rapidity and accuracy. It gradually increased from 0.505 to 0.610 S/m during 42 days at 5 °C and promptly increased from 0.708 to 1.195 S/m during 30 days at 20 °C, proving the prospect of electrical conductivity for rapid detection of the MF during storage [6]. However, the electrical conductivity measurement system is costly which limited the further popularization to the common people.

Intelligent labels can convey instant freshness information to consumers via visual color changes [7]. In fact, the bacteria in milk ferment carbohydrates to produce lactic acid which causes a gradual and slight decrease in pH [5,8]. Thus, the labels to assess MF via changes to pH are preferred. Ma et al. developed an intelligent film to monitor MF composed of tara gum, cellulose nanocrystals, and grape skin extract, where the color of the intelligent film changed from yellow to darker yellow and red as an indicator of milk spoilage [9]. In addition, Li et al. developed a colorimetric active double-layer film to evaluate MF that was composed of oxidized sodium alginate, carboxymethyl chitosan, and an extract of *Oxalis triangularis* ssp. papilionacea, where the composite color changed from grass green to light brown over time as an indicator of milk spoilage [1]. However, those indicator films are limited by the indistinguishable color changes at milk pH values of 6.54 to 6.49.

Synthetic colorimetric indicators have also been developed that rapidly respond to changes in pH values as compared to natural indicators, including the bromocresol purple (BP) [10,11], which shifts color over a pH range of 5.2–6.8, close to the pH range of milk during the storage period. Accordingly, BP is a suitable candidate to monitor MF. BP has been embedded in a polymer-based film to monitor the freshness of various foods. Yimit et al. encapsulated BP in a hybrid intercalated silica sol-gel and polymethacrylic acid system for sensing gases released from mutton samples [10]. Zakaria et al. fabricated an alginate/Ca/BP composite film to monitor the spoilage of protein-based foods [12]. Noting that the color changes in this research were relatively slow due to the highly compact structure of the developed film. In addition, BP had been bound covalently to an ethylene-vinyl alcohol copolymer for detecting milk freshness. The results showed that the thickness of the sensors had a significant effect on the discoloration which could cause an imprecise indication [13]. As a result, a dimensionally stable and porous matrix is needed.

In this study, cellulose paper was selected as a support matrix based on relatively simple processing, abundance, and porosity [14,15]. The small size of BP inhibits interactions with cellulose which may result in a lower retention rate and decreased accuracy. Hence, a carrier that can form chemical bonds with cellulose and firmly anchor BP was developed using rice straw (RS), an abundant waste crop resource that contains large amounts of cellulose, hemicellulose, and lignin [16].

Around 509.87 million metric tons of RS are either burned, used to feed livestock, or discarded in landfills each year [17]. Our group previously reported successful modification of rice straw particles (RSPs) using mono-quaternaries to adsorb bromothymol blue (adsorption capacity of 95.81 mg/g) for monitoring the freshness of pork and fish [18].

Here, to improve sensitivity, bis-ammonium salt (N, N, N′, N′-tetramethyl ethylenediamine) was applied to modify RSPs for attaching BP as a sensor embedded in RS cellulose paper to monitor MF. The effects of the adsorbent dosage and adsorption time on the adsorption capacity of BP were evaluated. Morphological changes to the samples were observed by scanning electron microscopy (SEM), while the functional groups and crystal structures were identified by Fourier transform infrared (FTIR) spectroscopy, X-ray photoelectron spectroscopy (XPS), and X-ray diffraction (XRD). In addition, the mechanical properties, sensitivity to pH and acetic acid (HAc), ventilation capacity, and thermal stability were evaluated for the practical application of the intelligent cellulose-based label to monitor MF.

## 2. Materials and Methods

### 2.1. Materials

Rice straw was harvested from Wuchang (Harbin, China). Sodium hydrate (NaOH), N, N, N′, N′-tetramethyl ethylenediamine (C_6_H_16_N_2_), and bromocresol purple (BP) were purchased from Shanghai Macklin Biochemical Co., Ltd. (Shanghai, China). Epichlorohydrin (C_3_H_5_ClO) and dimethyl carbonate (C_3_H_6_O_3_) were obtained from Shanghai Aladdin Biochemical Technology Co., Ltd. (Shanghai, China).

### 2.2. Modification of RSPs

Waste RS was crushed with a grinder (QJ-10B, Shanghai, China) and passed through the sieve with a pore size of 0.125–0.150 mm. Then, 5 g of the filtered RS was soaked in 125 mL of 20% NaOH solution and stirred for 2 h. Afterward, 62.5 mL of the NaOH solution was mixed with 60 mL of epichlorohydrin and stirred at a speed of 500 r/min for 6 h at 65 °C. After that, the rice straw particles crosslinked with epichlorohydrin were separated, rinsed with ethanol to remove excess epichlorohydrin, and added to 50 mL of N, N, N′, N′-tetramethyl ethylenediamine while stirring for 3 h at 65 °C. Afterward, the suspension was filtered and washed with ethanol (~150 mL) and water (~150 mL). Then, 40 mL of dimethyl carbonate was added, and the mixture was stirred at a speed of 500 r/min for 1 h at 80 °C. Finally, the prepared BC-RSPs were thoroughly washed with water until the pH of the filtrate was around 7.

### 2.3. Adsorption Experiments

The effects of adsorbent dosage and adsorption time on adsorption capacity in 0.001 M alkaline solution (100 mg/L) were evaluated. Briefly, different amounts of BC-RSPs were added to 50 mL of BP/NaOH solution while stirring at 150 rpm at various temperatures until the adsorption reached an equilibrium. After filtration, the absorption of the supernatant was measured at 375.8 nm with an ultraviolet-visible spectrophotometer (UV-2600; Shimadzu Corporation, Kyoto, Japan) and a standard curve of BP in 0.001 M NaOH solution was generated (y = 0.0159x + 0.0184; R^2^ = 0.995). The adsorption capacity (Q_t_) was calculated as

Q_t_ = [(A_i_ − A_t_)**/**m] × V,

where A_i_ and A_t_ (mg/L) are the concentrations of BP in the NaOH solution at the initial and final times (i and t, respectively), m (g) is the amount of BC-RSPs, and V (mL) is the volume of the BP/NaOH solution.

### 2.4. Preparation of the Cellulose-Based Label

The intelligent cellulose-based label was prepared via the traditional papermaking method. Specifically, the rice straw cellulose fibers were cut into different beating degrees (30, 40, and 50 °SR), then mixed with BP-BCRPs in different mass ratios (9:1, 8:2, 7:3, and 6:4) in a speed of 700~800 r/min using a fiber disintegrator (GBJ-A, Jinan, China). Finally, they self-assembled into the cellulose-based label with a basis weight of 60 g/m^2^ assisted by a paper forming machine (ZQJ1-B-II), and dried at 105 °C for 10 min.

### 2.5. Characterizations

The microstructure was observed using Scanning electron microscopy (SEM, Apreo S HiVac, Thermo Scientific, MA, America. The functional group changes were researched via the Fourier transform infrared spectrometer (Nicolet iS50,Thermo fisher, Shanghai, China) at a scanning range of 500–4000 cm^−1^. The elemental analysis was conducted by X-ray photoelectron spectroscopy (XPS, AXIS Ultra DLD, Kratos, Manchester, UK). The crystalline structure was studied using X-ray diffraction (XRD, D/max 2200,Rigaku Corporation, Akishima-shi, Tokyo, Japan).

### 2.6. Thermal Stability

The tested sample mass was 8~10 mg and was cut into pieces with a size of ~4 mm^2^. The thermal stability of that was conducted via a thermogravimetric analyzer (TGA Q500: TA Instruments**,** New Castle, DE, USA) from 25 to 600 °C at intervals of 10 °C/min.

### 2.7. Mechanical Properties

The intelligent label was cut into sections of 15 × 80 mm. The thickness of the sections was measured using a film thickness tester (DC11ZXBS; Mitutoyo, Kanagawa, Japan). The tensile strength (TS) and elongation at break (EB) were assessed with an auto tensile tester (PARAM**^®^** XLW (PC); Labthink Instruments Co., Ltd., Jinan, China) at a strain rate of 300 mm/min.

### 2.8. Ventilation Capacity

The intelligent label with an area of 20 cm^2^ was prepared for testing the ventilation capacity (air permeability) which was conducted with a numerical air permeability tester (YG461E; Inteke Instrument Co., Ltd., Shenzhen, China), and the average of three parallel samples were obtained from each group.

### 2.9. Response to pH

The intelligent label was cut into circles (diameter, 15 mm) which were immersed in pH buffers for 30 s and then imaged. The chromaticity parameters were measured using a hand-held colorimeter (CM-2600d; Konica Minolta, Tokyo, Japan). The difference in color (ΔE) was calculated as follows

ΔE = [(L* − L_0_*)^2^ + (a* − a_0_*)^2^ + (b* − b_0_*)^2^]^1/2^

where L, a, and b are the chromaticity parameters of initial label, and L_0_, a_0_, and b_0_ are the chromaticity parameters after discoloration.

### 2.10. Response to Acetic Acid (HAc)

The intelligent label (size, 1 × 1 cm) was adhered inside a container with a humidity of 53%. HAc at different volumes was injected using a syringe to produce an acidic environment. The discoloration time of the intelligent label was recorded, and the chromaticity parameters were measured using a hand-held colorimeter after 10 min.

### 2.11. Monitoring of MF

Fresh milk was purchased from a local supermarket and 10 mL aliquots were used for measurement of pH and acidity at regular intervals. The surplus milk was stored at 40 °C. The intelligent label was immersed in milk for 30 s and color changes were assessed. Meanwhile, the corresponding chromatic parameters were measured with a colorimeter.

### 2.12. Statistical Analysis

The samples used for the performance testing were prepared in triplicates, and the tested results were presented as their means ± standard deviations and analyzed using SPSS software (v17.0; Chicago, IL, USA) with a significant difference at *p* < 0.05.

## 3. Results and Discussion

### 3.1. Preparation of Cationic RSPs (BC-RSPs) and Adsorption of BP

**Figure 1:**
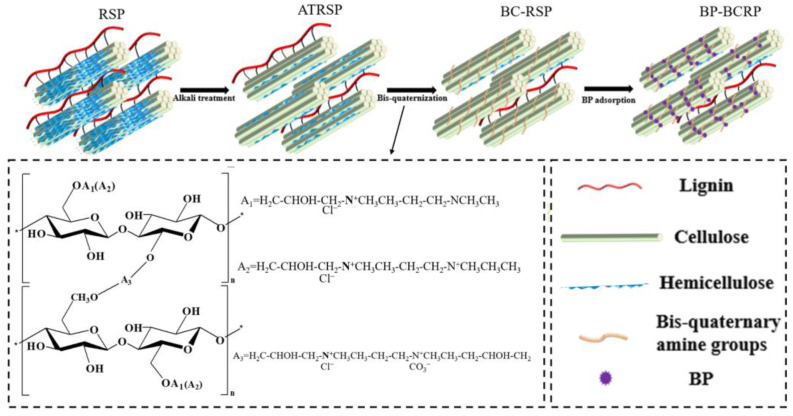
Preparation mechanism of bromocresol purple (BP) attached rice straw particles (RSP).

The preparation of cationic RSPs is illustrated in Figure 1. The structural network of RSPs is mainly formed by cellulose, lignin, and hemicellulose. Hemicellulose combines with celluloses via hydrogen bonds to support lignin which acts as a natural adhesive. Alkali treatment removed most hemicellulose, lignin, and wax, thereby effectively improving the accessibility of cellulose [19,20]. To prepare cationic RSPs, cationic groups were introduced to the alkali-treated RSPs by crosslinking of N, N, N′, N′-tetramethyl ethylenediamine with epichlorohydrin [21] (Figure 1).

**Figure 2:**
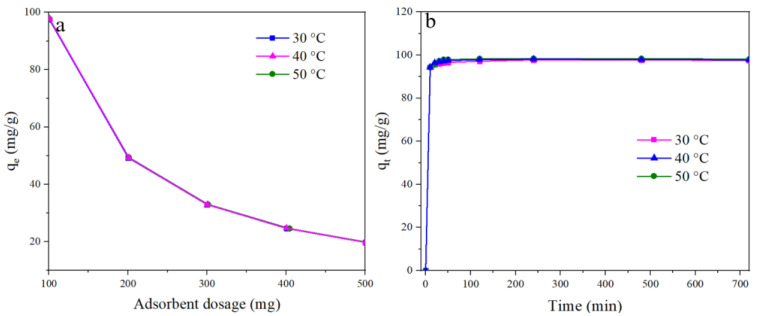
The effects of the adsorption dosage (**a**) and the adsorbent time (**b**) on the adsorption capacity.

BP is negatively charged in an alkali solution and was attached to the positively charged BC-RSPs via electrostatic forces. As seen in Figure 2, an evaluation of the effects of the adsorbent amount and adsorption time on the adsorption capacity for BP found that the adsorption temperature had no significant effect on adsorption capacity. As the adsorbent dosage increased from 100 to 500 mg, the equilibrium adsorption capacity (q_e_) decreased from 97.68 to 19.87 mg/g which might be due to the limited amount of BP molecules attached to more sites, letting the adsorption capacity decrease. The adsorption capacity rapidly increased with the adsorption time. Then, the growth rate slowed until the adsorption capacity reached an equilibrium. Further increases in the adsorption time had no significant effect on the adsorption capacity because the attachment sites had been saturated [22].

### 3.2. SEM Images of Fibers

**Figure 3:**
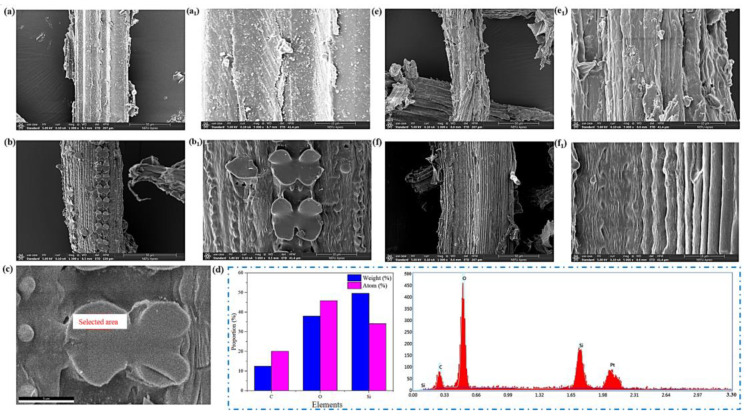
SEM images of rice straw particles (RSPs) (**a**,**a_1_**); alkali treated RSPs (ATRSPs) (**b**,**b_1_**,**c**); bis-quaternized RSPs (BC-RSPs) (**e**,**e_1_**); BP attached BC-RSPs (BP-BCRPs) (**f**,**f_1_**); EDS spectrum of ATRSP (**d**).

Micromorphological changes were assessed by SEM. As shown in Figure 3a,a_1_, the surface of pristine RSPs cloaked in a layer of wax and SiO_2_ was rough without obvious fiber strips [23]. While the surface of alkali-treated RSPs (ATRSPs) exhibited parallel and neatly arranged fiber bundles (Figure 3b,b_1_) because alkali opened the obstinate structure of the RSPs by removing most of the lignin, hemicellulose, and wax layer, effectively increasing accessibility of the cellulose [24]. In addition, there were four-leaf grass-like structures (Figure 3c) on the ATRSP surface which were identified as residual silicon by energy-dispersive X-ray spectroscopy (Figure 3d). After bis-quaternization, the BC-RSPs exhibited uniform and shallow creases, while the four-leaf grass-like silicon had disappeared (Figure 3e,e_1_) which may be owing to the further removal of residual silicon in the alkaline environment during bi-quaternization. In Figure 3f,f_1_, SEM images of BP-BCRPs showed more obvious shallow creases because adsorption was also conducted in an alkali solution.

### 3.3. Preparation of the Cellulose-Based Label

**Figure 4:**
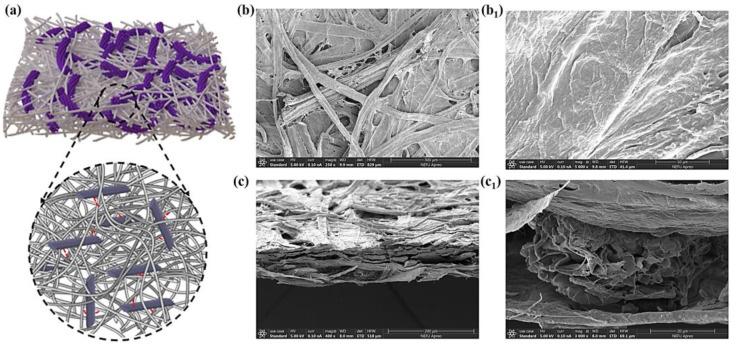
The three-dimensional structure of cellulose-based label (**a**); SEM images of the label surface (**b**,**b_1_**) and cross section (**c**,**c_1_**).

The RS cellulose fibers were cut into different beating degrees (30 °SR, 40 °SR, and 50 °SR) and then mixed with BP-BCRPs (adsorption capacity, 97.68 mg/g) to form the intelligent cellulose-based label via hydrogen bonding (Figure 4a). As shown in Figure 4b, rod-shaped BP-BCRPs were entangled among the cellulosic fibers to construct a porous three-dimensional structure. Cross-section SEM images (Figure 4c) revealed relatively loose stacking of the layers. Capillary action of the liquid often occurred along the gaps of cellulose paper [25] that would contribute to the point-to-surface response of the label.

### 3.4. Chemical Structures

**Figure 5:**
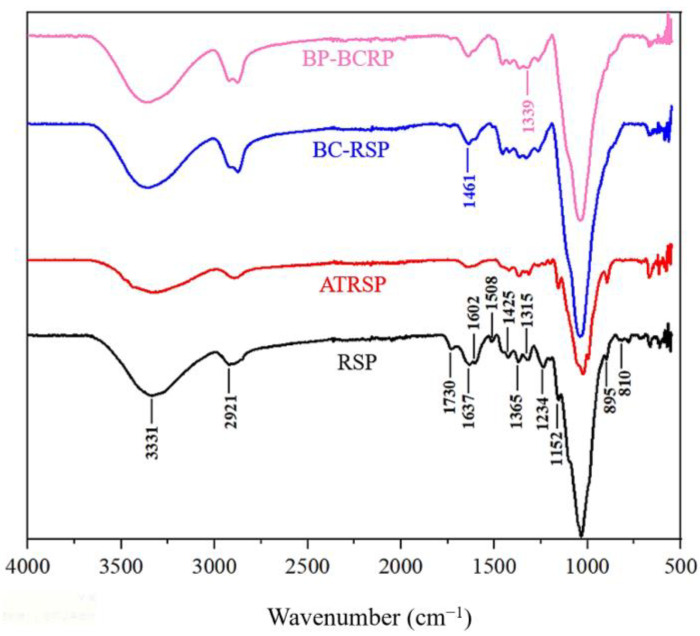
FTIR spectrums of RSP, ATRSP, BC-RSP, and BP-BCRP.

XPS and FTIR spectrums were used to elucidate the reaction mechanism during modification. From Figure 5, the FTIR spectrum of RS included characteristic peaks of cellulose, hemicellulose, and lignin. For cellulose, the peaks at 1637, 1365, 1315, 1152, and 895 cm^−1^ represent the adsorption of O–H and conjugation of C–O in polysaccharides, C–H deformation, CH_2_ wagging, C–O–C asymmetric stretching, and CH deformation of beta-glycosidic linkages, respectively. For hemicellulose, the bands at 1730, 1461, and 810 cm^−1^ ascribe to C=O of the ester and acetyl groups, asymmetry of -CH_3_ and CH_2_, and vibration of mannan, respectively. For lignin, the peaks at 1602, 1508, 1461, 1425, and 1234 cm^−1^ are contributed to C=O stretching conjugated to the aromatic ring and carboxylic groups, C=C stretching of the aromatic ring and C=O bond vibrations of the extractive compounds, C–H asymmetric deformation of methoxyl, aromatic skeletal vibrations, and OH vibrations of the guaiacyl ring, respectively [26,27,28]. After alkali treatment, the intensity of the absorption peaks of lignin and hemicellulose was greatly reduced, indicating that the alkali treatment had effectively removed lignin and hemicellulose and opened the structure. After bis-quaternization, the absorption peaks of lignin and hemicellulose had completely disappeared, and a new peak at 1461 cm^−1^ of the quaternary ammonium group confirmed successful bis-cationization [29]. In the FTIR spectrum of BP-BCRPs, the peak at 1339 cm^−1^ [30], denoting the presence of sulfonate groups, indicated successful attachment of BP.

**Figure 6:**
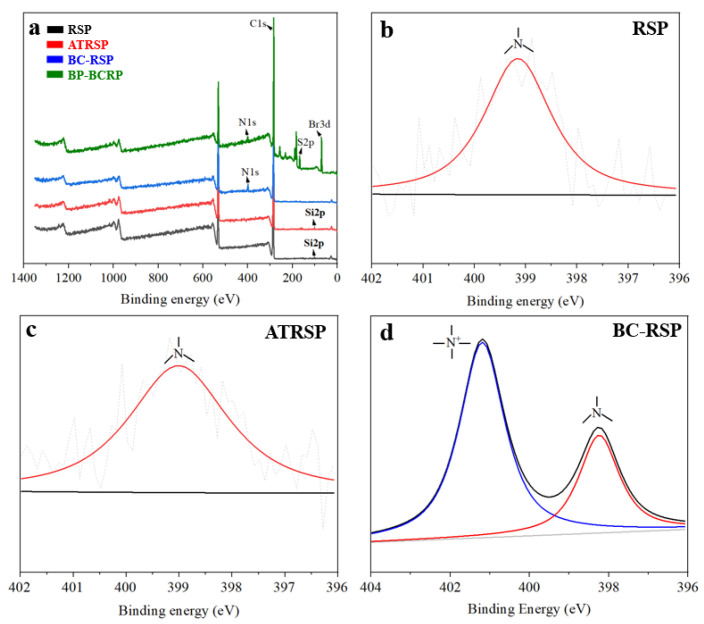
XPS spectrums (**a**); deconvoluted spectrums of N1s in RSP (**b**); ATRSP (**c**); and BC-RSP (**d**) (the N peaks represented in pink and blue were from crude protein of rice straw and bis-quatenary ammonium salts, respectively).

XPS was used to determine element changes during the modification. As shown in Figure 6a, RSPs mainly contained C and O (from cellulose, hemicellulose, and lignin), Si (from SiO_2_), and N which may derive from the crude protein of RS (Figure 6b). For ATRSP, the peak intensity of Si decreased due to the alkali treatment, agreeing with the results of SEM images. In addition, the N1s peak at 399.02 eV showed that the alkali had no influence on crude protein (Figure 6c). After bis-quaternization, the peak intensity of Si completely disappeared. The peak of N1s in BC-RSP could be fitted with two peaks at 399.64 eV and 402.28 eV which corresponded with nitrogen in the crude protein and bis-quaternary ammonium groups, respectively [21], further confirming the successful grafting of quaternary ammonium cations onto the RSPs (Figure 6d).

### 3.5. Crystalline Structure and Thermal Stability

**Figure 7:**
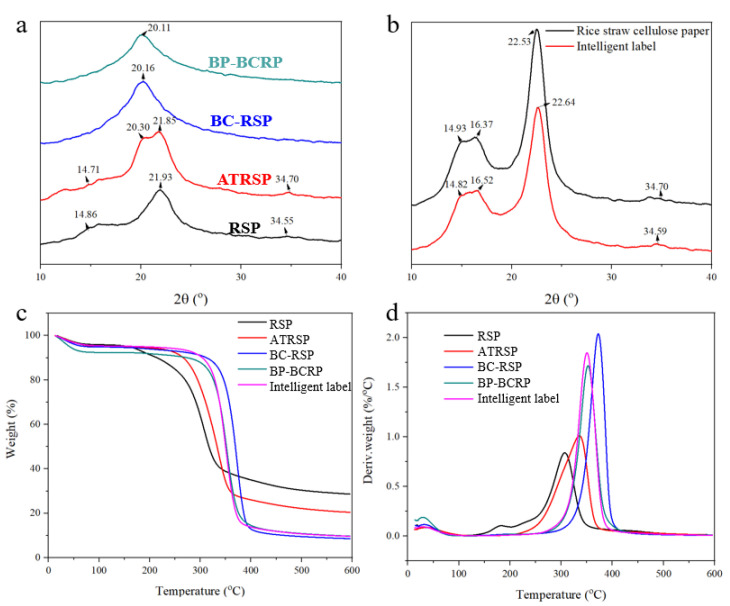
XRD patterns of cellulose modification (**a**) and the intelligent label (**b**); TGA (**c**) and DTG (**d**) curves of that.

XRD, thermogravimetric analysis (TGA), and difference thermogravimetry (DTG) were used to assess the crystalline structure and thermal stability. As shown in Figure 7a, RSPs exhibited a typical cellulose I structure consisting of 2θ = 14.86° (101), 2θ = 21.93° (002), and 2θ = 14.86° (040) [31]. The new peak at 2θ = 20.30° after alkali treatment was ascribed to the cellulose II structure [32]. Alkali treatment destroyed the original ordered crystal structure of RSPs and the molecular chain was rearranged to form a new ordered structure. After quaternization, the crystal structure of cellulose completely shifted from type I to II because bis-quaternization also occurred under alkaline conditions. The crystal structure of the intelligent label (Figure 7b) exhibited a typical cellulose I crystal form due to the higher proportion of RS cellulose.

TGA and DTG curves of RSPs, ATRSPs, BC-RSPs, BP-BCRPs, and the intelligent label are shown in Figure 7c,d. Thermal degradation of RSPs included three stages. From room temperature to 100 °C, the decrease in weight was due to the evaporation of free and adsorbed water. The second stage from 150 to 210 °C was characterized by pyrolysis of hemicellulose and partial lignin. The third stage from 210 to 350 °C was ascribed to cellulose and remaining lignin. After alkali treatment, the pyrolytic peak of hemicellulose disappeared, confirming the removal of almost all hemicellulose. The maximum pyrolysis peak of cellulose shifted to a higher temperature of 372.50 °C after bis-quaternization, likely due to the removal of the lignin and hemicellulose, as the crystalline structure of cellulose gradually changed from type I to II along with denser stacking of the cellulose. During the modification, the residual weight decreased from 28.69% to 8.59%. For BP-BCRPs and the intelligent label, the residual weight reached 9.80% due to the adsorption of BP. TGA and DTG analysis confirmed improved thermal stability of BC-RSPs due to the removal of the lignin and hemicellulose. Notably, the change to the crystal structure improved thermal stability [33].

### 3.6. Air Permeability and Mechanical Properties

The air permeability and mechanical properties of the cellulose-based labels with different beating degrees and BP-BCRPs addition ratios were tested. As shown in Table 1, air permeability decreased from 73.22 to 15.01 mm/s and TS increased from 7.38 to 9.87 MPa as the beating degree increased from 30 °SR to 50 °SR, as a higher beating degree improved fibrillation to form more hydrogen bonds and a denser network structure. As shown in Table 2, air permeability increased from 3.76 to 15.01 mm/s, while TS had decreased from 18.25 to 9.87 MPa as the proportion of BP-BCRPs increased from 10% to 40%, likely because the BP-BCRPs with a size of 100–120 mesh were used as the response fillers within the three-dimensional network formed by the self-assembly of long RS cellulose fibers which cannot be replaced by the interweaving of BP-BCRPs. Thus, the three-dimensional structure was loosened as the proportion of BP-BCRPs increased from 10% to 40%.

### 3.7. Responses to pH and HAc

The response to pH is crucial to evaluate the application of an intelligent label which exhibited yellow at pH = 3~4, yellow-green at pH = 5, light cyan at pH = 5.5, dark cyan at pH = 6, baby blue at pH = 6.5, blue at pH = 7, blue-purple at pH = 8, and purple at pH = 9 (Table 3). The chromaticity parameters of the cellulose-based label in different pH buffers are shown in Table 3. The a* value decreased from −1.35 to −3.42 as the pH value was increased from 3 to 6.5 which resulted in a shift of the color of the label to green on the red-green axis. An increase in the a* value from −1.32 to 2.64 with the further increase of pH from 7 to 9 resulted in the color changing to red on the red-green axis. The b* value decreased from 16.19 to −22.64 as the pH value increased from three to nine, showing the color of the intelligent label shifted to blue on the yellow-blue axis. The color change can be observed unaided at ΔE > 5. As shown in Table 3, ΔE was >5 at all pH values, confirming that the indicator label has the potential to monitor MF with highly saturated color changes [34].

**Figure 8:**
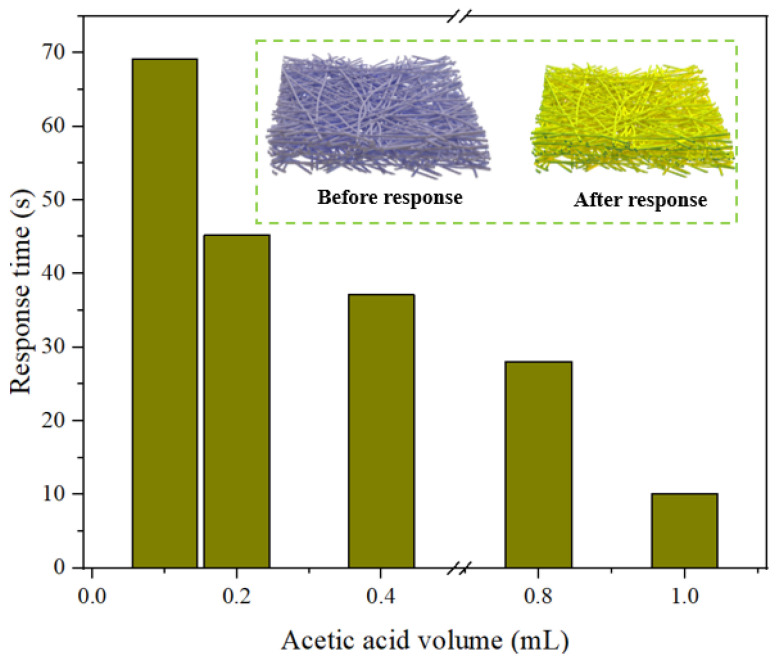
Response time of the intelligent label to different acetic acid volumes.

The responses of the intelligent label to HAc are shown in Figure 8, and the color parameters are shown in Table 4. The response time decreased from 69.17 s to 10.12 s as the volume of HAc increased from 0.1 mL to 1.0 mL because the higher volume brought more HAc molecules to gather on the label surface, resulting in the label color shifting from blue to yellow. In addition, it is noted that the ΔE value gradually increased from 19.05 to 27.61 when the HAc volume increased, showing the label color change was more obvious with a higher HAc volume due to more H^+^.

### 3.8. Monitoring of MF

**Figure 9:**
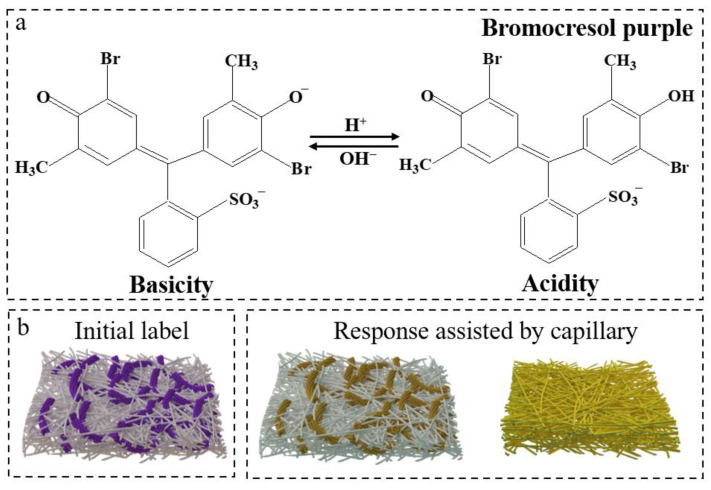
Response mechanism of bromocresol purple in basic and acidic condition (**a**); the discoloration mechanism of the indicator label in the milk (**b**).

In this study, the intelligent label was used to monitor MF in response to changes in pH values of 5.2–6.8. Acidity and pH are often used to evaluate MF. As shown in Table 5, the acidity, lactic acid content, and pH of fresh milk were 17.24 °T, 0.16%, and 6.42, respectively, as the initial label color was blue. After 4 h, the acidity and lactic acid content increased to 18.81 °T and 0.17%, while pH decreased to 6.37, and the corresponding label color shifted from blue to cyan. After 6 h, the milk acidity and lactic acid content reached 19.8 °T and 0.18%, close to the threshold of deterioration. At this time point, the color of the BP-BCRPs began to gradually yellow, and the cellulose-based matrix was also yellow in color due to the unique capillary action. The change in color acts as a reminder to the consumer of potential spoilage to avoid waste. After storing for 8 h, floccule formation was observed in the milk as the acidity and lactic acid content increased to 22.77 °T and 0.2%, indicating the milk was completely spoiled. The corresponding label had changed to a distinguishable yellow. The response mechanism of BP-BCRPs to MF is illustrated in Figure 9. After immersing the label into milk, BP-BCRPs within the label sensed the pH changes around with a color change from blue-purple to yellow. The liquid spread throughout the gaps via capillarity action. These results confirmed that the intelligent label can track MF in real time and alert the consumer when the milk is on the verge of spoilage.

## 4. Conclusions

In this study, a rapid, precise, colorimetric, cellulose-based label was prepared to monitor MF. RSPs were treated using alkali to open obstinate structures to improve the accessibility of the cellulose which was grafted with bis-quaternary ammonium salt groups to attach BP via electrostatic bonding. The maximum adsorption capacity of BP was 97.56 mg/g. The intelligent label was composed of BP-BCRPs embedded into RS cellulose paper by hydrogen bonds. The results of FTIR and XPS confirmed successful grafting of the bis-quaternary ammonium salt groups and adsorption of BP. The results of XRD and TGA confirmed the improved thermal stability of RSPs by bis-quaternization due to the shift of the crystalline form from type I to type II. More importantly, the intelligent label exhibited excellent responses to changes in pH values. For monitoring of MF, the yellow color of the intelligent label serves to warn the consumer that the milk is close to spoilage to avoid waste. Overall, the results of this study confirm that the prepared cellulose-based intelligent label is suitable to monitor MF.

## Figures and Tables

**Table 1:** Air permeability of the intelligent label prepared with different beating degrees.

Beating Degrees (°SR)	Air Permeability (mm/s)	σ (N)	TS (MPa)
30	73.22 ± 0.19 ^a^	18.88 ± 0.05 ^b^	7.38 ± 0.08 ^a^
40	40.16 ± 0.37 ^a^	20.18 ± 0.13 ^a^	8.40 ± 0.21 ^a^
50	15.18 ± 0.14 ^b^	21.63 ± 0.24 ^a^	9.12 ± 0.18 ^a^

Data are mean ± standard deviation, and mean within different letters within the same column are significant different (*p* < 0.05).

**Table 2:** Air permeability and mechanical properties of the intelligent label containing different BP-BCRP ratios.

BP-BCRP Ratios (%)	Air Permeability (mm/s)	σ (N)	TS (MPa)
10	3.76 ± 0.12 ^c^	42.54 ± 0.20 ^b^	18.25 ± 0.15 ^c^
20	5.04 ± 0.24 ^b^	32.95 ± 0.14 ^c^	14.27 ± 0.19 ^a^
30	5.91 ± 0.10 ^a^	29.73 ± 0.01 ^a^	13.63 ± 0.13 ^a^
40	15.01 ± 0.26 ^a^	21.97 ± 0.13 ^b^	9.87 ± 0.07 ^a^

Data are mean ± standard deviation, and mean within different letters within the same column are significant different (*p* < 0.05).

**Table 3:** Chromaticity parameters of pH response.

pHs	L*	a*	b*	ΔE	Color Changes
3.0	79.32 ± 0.05 ^a^	−1.35 ± 0.03 ^a^	16.19 ± 0.01 ^a^	17.06 ± 0.02 ^a^	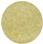
4.0	77.73 ± 0.01 ^b^	−1.92 ± 0.02 ^c^	12.02 ± 0.01 ^c^	13.54 ± 0.08 ^b^	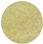
5.0	74.04 ± 0.02 ^a^	−2.71 ± 0.01 ^a^	10.82 ± 0.03 ^c^	14.42 ± 0.01 ^c^	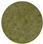
5.5	73.07 ± 0.03 ^b^	−4.08 ± 0.07 ^a^	−2.24 ± 0.02 ^b^	9.47 ± 0.01 ^a^	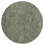
6.0	73.55 ± 0.05 ^c^	−3.11 ± 0.01 ^c^	−3.13 ± 0.07 ^a^	8.90 ± 0.04 ^a^	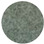
6.5	78.2 ± 0.05 ^b^	−3.42 ± 0.03 ^a^	−5.88 ± 0.01 ^c^	6.29 ± 0.03 ^a^	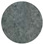
7.0	69.01 ± 0.04 ^a^	−1.32 ± 0.03 ^a^	−14.90 ± 0.06 ^a^	20.01 ± 0.05 ^b^	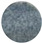
8.0	65.70 ± 0.02 ^b^	0.86 ± 0.02 ^c^	−22.10 ± 0.09 ^a^	27.72 ± 0.01 ^a^	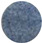
9.0	62.90 ± 0.01 ^b^	2.64 ± 0.04 ^a^	−22.64 ± 0.06 ^a^	30.13 ± 0.02 ^a^	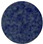

Data are mean ± standard deviation, and mean within different letters within the same column are significant different (*p* < 0.05).

**Table 4:** Chromaticity parameters of HAc response.

HAc Volume (mL)	L*	a*	b*	ΔE	Color Changes
0.1	79.87 ± 0.12 ^b^	−1.53 ± 0.08 ^a^	17.55 ± 0.07 ^a^	19.05 ± 0.10 ^a^	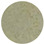
0.2	80.19 ± 0.11 ^a^	−1.76 ± 0.06 ^a^	20.00 ± 0.07 ^c^	21.46 ± 0.16 ^b^	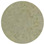
0.4	82.30 ± 0.15 ^a^	−1.73 ± 0.06 ^a^	22.45 ± 0.01 ^a^	23.82 ± 0.18 ^a^	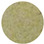
0.8	83.11 ± 0.18 ^a^	−1.56 ± 0.02 ^a^	23.44 ± 0.04 ^c^	24.83 ± 0.09 ^a^	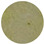
1.0	84.80 ± 0.17 ^b^	−1.62 ± 0.09 ^b^	26.11 ± 0.06 ^b^	27.61 ± 0.24 ^a^	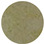

Data are mean ± standard deviation, and mean within different letters within the same column are significant different (*p* < 0.05).

**Table 5:** The pH, acidity, and lactic acid content of milk during storage and the corresponding discolorations of the indicator label.

Milk Storage Time (h)	pH	Acidity (°T)	Lactic Acid Content (%)	Discolorations
0	6.42	17.24	0.16	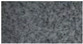
4	6.37	18.81	0.17	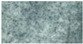
6	6.21	19.80	0.18	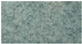
8	5.74	22.77	0.20	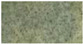

## Data Availability

The data are available from the corresponding author.

## Abbreviations

Rice straw particles (RSPs); Alkali treated RSPs (ATRSPs); Bis-quaternized RSPs (BC-RSPs); Bromocresol purple (BP); BP attached BC-RSPs (BP-BCRPs); Milk freshness (MF).
